# Estimating qualification and factors associated with third-line antiretroviral therapy referral in the Western Cape

**DOI:** 10.4102/sajhivmed.v22i1.1184

**Published:** 2021-01-28

**Authors:** Sadiyya Sheik, Bart Willems

**Affiliations:** 1 Department of Global Health, Faculty of Health Systems and Public Health, Stellenbosch University, Cape Town, South Africa

**Keywords:** third-line ART, resistance, public health, HIV, Western Cape

## Abstract

**Background:**

South Africa’s antiretroviral therapy (ART) programme is the largest globally and the universal test-and-treat policy is expected to increase the numbers on ART. This may have implications for treatment failure rates implying a greater future need for third-line regimens. South Africa initiated a third-line programme in 2013. However, there is little evidence quantifying the third-line need in this setting and the programme itself has not been formally evaluated.

**Objectives:**

The study evaluated the third-line ART referral process in the Western Cape.

**Method:**

Routinely collected data were analysed to derive an estimate of patients meeting criteria for third-line referral and compared with patients who were referred. Factors associated with referral were identified.

**Results:**

In the study period, 947 patients met criteria for third-line referral and 167 patients were referred. Comparison revealed a poor overlap of only 42 patients. In multivariate analysis, factors associated with referral included receiving care at a hospital rather than a primary healthcare facility (adjusted odd ratios [aOR] = 2.15, 95% confidence interval [CI] 1.1–4.2), a higher number of viral load [VLs] ≥ 1000 copies/mL whilst on a protease inhibitor (PI) (aOR = 1.2, 95% CI 1.01–1.42) and a greater number of years on a PI (aOR = 1.25, 95% CI 1.07–1.46). Patients with a 6-month gap in dispensing were less likely to be referred (aOR = 0.37, 95% CI 0.17–0.81).

**Conclusion:**

This study adds to a limited body of knowledge regarding third-line ART programmes. The findings indicate missed opportunities for and inappropriate referral of patients. Factors associated with referral were largely health system related. Clinician awareness and compliance with referral remain unknown and may be contributory.

## Introduction

Human immunodeficiency virus (HIV) is a major contributor to morbidity and mortality in South Africa and remains a prominent part of the country’s burden of disease landscape – despite gains in life expectancy linked to large scale roll-out of antiretroviral therapy (ART).^1^ Public sector ART has been available in South Africa for more than a decade and there are more than 5.2 million people receiving ART in the country^2^ making the South African ART programme the largest in the world.

In 2016, South Africa adopted a universal test-and-treat policy, making ART accessible to all people living with HIV regardless of CD4 count. This has increased the number of South Africans on ART but may have additional implications for rates of treatment failure and drug resistance as a result of increasing exposure to ART. In the future, there may be an amplified need for second and third-line ART in the country.

The South African National Department of Health (NDoH) initiated the world’s first public sector third-line ART access programme in April 2013. Unlike the first and second-lines, which are pre-defined regimens following a public health approach, there is no standard third-line regimen. The choice of individual drugs included in a third-line regimen is made on a case-by-case basis considering the patient history and results of genotype resistance testing. Third-line therapy can only be accessed via a referral process in which clinicians motivate to a central third-line committee first to obtain genotype testing for patients who meet the defined referral criteria and subsequently third-line ART depending on the outcome of the genotype testing.

Third-line programmes are in their infancy worldwide and there is little evidence quantifying the need for third-line therapy in the South African setting. In addition, the referral process by which third-line ART is accessed has not been formally evaluated and predictors of referral are not known. Thus, there exists a critical need to evaluate third-line ART programmes in this setting. We evaluated a third-line ART referral process in the Western Cape province of South Africa by estimating qualification for referral to the provincial third-line ART committee, comparing the estimate with actual referrals and identifying factors associated with referral.

## Research methods and design

### Setting

The study was conducted in the Western Cape province of South Africa. There is one metropolitan and five rural districts in the Western Cape. Approximately two-thirds of the population reside in the Cape Town Metro District. In 2015, the antenatal HIV prevalence was 17.6% in the province and district prevalence varied from 11.6%^3^ in the Central Karoo to 18.9% in the Cape Town Metro. In 2015, approximately 60% (180 769) of the estimated HIV-infected population were on ART.^4^

### Western Cape third-line antiretroviral therapy referral criteria and process

Clinicians motivate to a provincial third-line committee for genotype testing and third-line ART for a defined group of patients who are failing second-line therapy. A panel of experts reviews each case to decide whether genotype resistance testing is warranted. If genotype testing is warranted, the panel then makes recommendations regarding the need for third-line therapy informed by a resistance score derived from the genotype results. Both genotype testing and third-line therapy are not routinely available outside of this access programme.

Adult patients (15 years and older) may be referred to the third-line ART committee if they have been receiving a protease inhibitor (PI) based regimen for ≥2 years and have virological non-suppression – defined as three viral loads ≥ 1000 copies/mL at least 8–12 weeks apart.^5^ In addition, good adherence should be verified objectively by pharmacy refill records. Clinicians are required to complete a formal application form, collating the patient’s clinical history and an adherence assessment form, which is completed together with the patient (see Online Appendix). Clinicians submit the necessary documentation via email and a decision regarding genotype testing and third-line therapy is made based on this documentation.

### Study design

**FIGURE 1:**
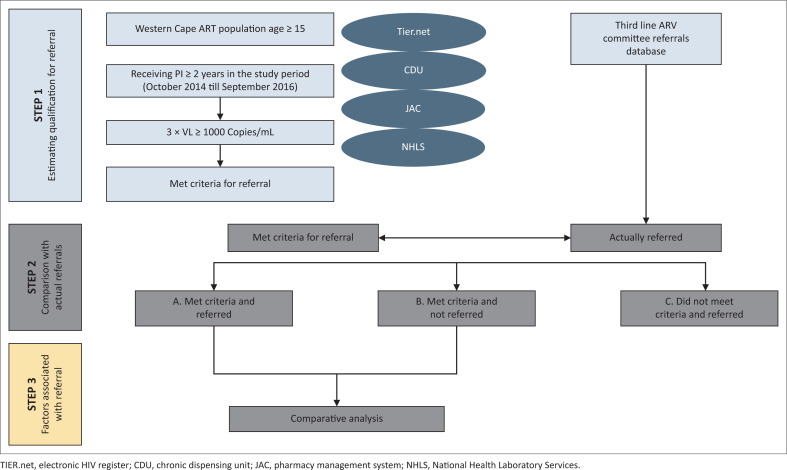
Graphic representation of study design.

The study was designed in three-steps in relation to the primary objectives of the research. Figure 1 is a graphic representation of the three-step study design and each of the steps is discussed in further detail.

#### Step 1: Estimating qualification for referral

The aim of the first step of the study was to identify all adult patients who met the criteria for referral to the third-line ART committee in the study period (01 October 2014–30 September 2016). Criteria for referral to the committee included: age ≥ 15 years, receiving a PI-based regimen for at least 2 years and virological non-suppression defined as at least three viral load measurements of ≥ 1000 copies/mL (≥ log 3) 8–12 weeks apart.

Routinely collected data for patients aged 15 years and older receiving PI-based ART during the study period was requested from the newly established Provincial Health Data Centre (PHDC). The centre curates data from a number of stand-alone primary information systems and incorporates an inter-operability component that allows for the linkage of patient level data from each individual system using the Patient Master Index (PMI), which uniquely identifies individuals. Data were derived from four sources. Pharmacy records were obtained from three sources: TIER.net (an electronic register used for managing the HIV programme, JAC (a pharmacy management system) and the Chronic Dispensing Unit (CDU, a dispensing system used for chronic medication) as no one system was able to provide a complete dispensing history. JAC and CDU were considered preferred sources of dispensing data because the recording of data is automated and less prone to error than that derived from TIER.net, which requires manual capturing by information clerks. The fourth data source was the National Health Laboratory Service (NHLS) for data pertaining to viral load records. Data from these sources were cleaned and analysed to identify patients meeting the criteria for referral to the third-line ART committee in the study period.

#### Step 2: Comparison with actual referrals

In the second step of the study, a database of actual referrals to the third-line ART committee was obtained and analysed. The database was cleaned and records occurring outside the study period were excluded. This database was then matched with the estimate of patients meeting the criteria for referral (i.e. the output of Step 1) and based on the matching process, patients were classified into three categories: ‘Met Criteria and Referred’, ‘Met Criteria and Not Referred’ and ‘Did Not Meet Criteria and Referred’. In order to strengthen the matching process in the case of patients with multiple folder numbers, patient folder numbers from the database of actual referrals were first matched with an identification table provided by the PHDC, which identified a dominant identification number that grouped together multiple folder numbers from the same patient.

#### Step 3: Factors associated with referral

In the third step of the study, a comparative analysis of the groups ‘Met Criteria and Referred’ and ‘Met Criteria and Not Referred’ was undertaken with the aim of identifying factors associated with referral. Comparator variables were obtained from routinely collected data and two categories of variables were collected, that is, demographic (e.g. age, sex and facility type) and clinical (e.g. time on PI) data. Variables were selected based on findings from literature pertaining to switch from first to second-line therapy. Notably, certain patient-level and facility-level variables were excluded because of non-feasible data collection. (See discussion).

### Data analysis

Data were analysed using Stata version 13.1 (Statacorp Texas 2013) and a *p*-value of < 0.05 was considered statistically significant. Continuous variables were described using relevant summary statistics depending on the distribution of the data. Further analysis involved the testing of hypotheses to determine if each variable was independently associated with the outcome, that is, referral, using either chi-squared, Fischer’s exact or the Wilcoxon rank sum test depending on the distribution of data. Following univariate analysis, all variables were entered into a logistic regression model and a backward stepwise selection procedure was used to eliminate non-significant variables. Findings are presented as crude and adjusted odds ratios with 95% confidence intervals (CI).

We performed additional analyses on the group ‘Did Not Meet Criteria and Referred’. Whilst this group was not the focus of the study, further analysis of the patients in this group was relevant to a comprehensive evaluation of the third-line ART referral process. In addition, in the absence of a validation method for estimating qualification for referral, we conducted sensitivity analyses to determine the impact of varying certain data parameters on the estimate.

### Ethical consideration

Ethical approval to conduct the study was obtained from the Health Research Ethics Committee of Stellenbosch University (reference no. S16/09/162).

## Results

### Step 1: Estimating qualification for referral

**FIGURE 2:**
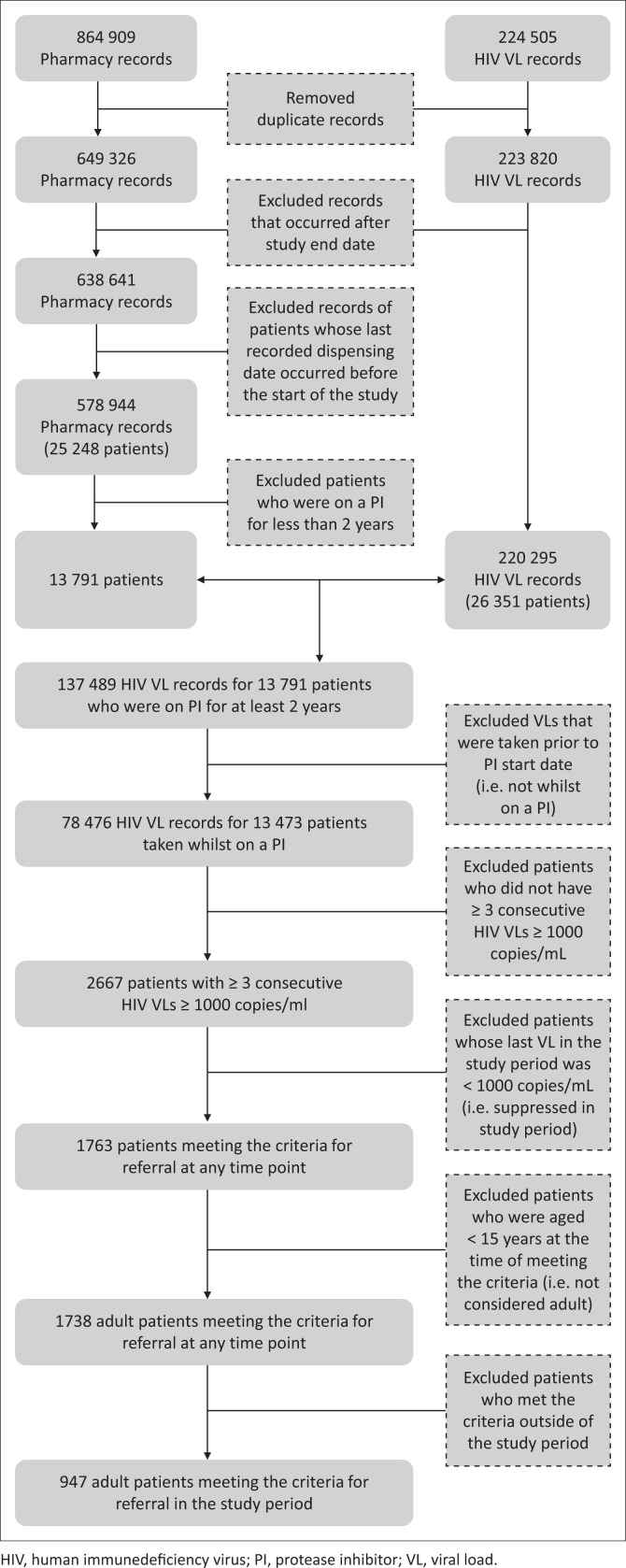
Estimating qualification for referral: Data cleaning and analysis process.

Of the 13 791 adult patients who were on a second-line regimen for at least 2 years, 947 (6.9%) met the criteria for referral to the third-line ART committee in the period 01 October 2014 to 30 September 2016. Figure 2 illustrates the data cleaning and analysis process undertaken to derive the estimate of patients meeting the criteria for referral. Table 1 provides descriptive characteristics for this cohort.

**TABLE 1:** Descriptive characteristics by group.

Variable	Met criteria for referral			Referred to the third-line committee	
	*N* = 947			*N* = 167	
	*n*	%	95% CI	*n*	%
Age, mean (s.d.)	38.6	9.9	-	38.4	9.1
Female sex	562	59.4	56.1–62.5	115	68.9
**Facility type**					
Hospital	252	26.6	23.8–29.5	77	46.1
PHC facility	695	73.4	70.5–76.2	90	53.9
**District**					
Cape Town Metro	677	71.5	68.5–74.3	136	81.4
Cape Winelands	133	14	11.9–16.4	22	13.2
Eden	77	8.1	6.5–10.1	7	4.2
Overberg	32	3.4	2.3–4.7	1	0.6
West Coast	18	1.9	1.1–3	1	0.6
Central Karoo	10	1.1	0.5–1.9	0	-
Rural	270	28.5	25.7–31.5	31	18.6
Time on PI in years, median (IQR)	3.2	2.6–4.2	-	-	-
Total number of high VLs in study period whilst on a PI†, median (IQR)	5	4–6	-	-	-
**Outcome of request for genotype**					
Approved	-	-	-	129	77.3
Rejected	-	-	-	14	8.4
Provided	-	-	-	14	8.4
Not recorded	-	-	-	10	6.0

PHC, primary healthcare; PI, protease inhibitor; IQR, interquartile range; CI, confidence interval, s.d., standard deviation.

† ,High VL defined as ≥ 1000 copies/mL.

### Step 2: Comparison with actual referrals

**FIGURE 3:**
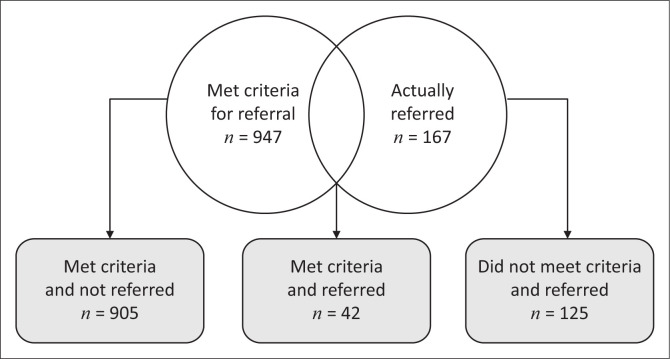
Overlap between patients who met the criteria and those who were referred.

A total of 167 adult patients were referred to the third-line ART committee in the period 01 October 2014 to 30 September 2016 (Table 1). Comparison of the two groups indicated that 42 patients met the referral criteria and were referred; 905 patients met the referral criteria and were not referred and 125 patients did not meet referral criteria but were referred (Figure 3 and Table 2).

**TABLE 2:** Descriptive characteristics by group.

Variable	Met criteria and referred			Met criteria and not referred			Did not meet criteria and referred		
	*N* = 42			*N* = 905			*N* = 125		
	*n*	%	95% CI	*n*	%	95% CI	*n*	%	95% CI
Age, mean (s.d.)	38.3	9.3	-	38.6	10	-	38.3	9.3	-
Female sex	27	64.2	48–78.4	537	59.3	56.1–62.6	88	70.4	66.6–78.2
**Year of meeting referral criteria**									
2014	3	7.1	1.5–19.5	114	12.6	10.5–14.9	n/a	-	-
2015	27	64.3	48–78.4	486	53.7	50.4–57	n/a	-	-
2016	12	28.6	15.7–44.6	305	33.7	30.6–36.9	n/a	-	-
Time (months) from meeting criteria to referral/end of observation period, median (IQR)	3.4	0.7–11.6	-	12.8	7.3–17.8	-	n/a	-	-
**Facility type**									
Hospital	18	42.9	27.7–59	234	25.9	23–28.8	58	46.4	37.4–55.5
PHC facility	24	57.1	41–72.3	671	74.1	71.2–77	67		44.5–62.6
**District**									
Cape Town Metro	36	85.7	71.5–94.6	641	70.8	67.7–73.8	98	78.4	70.2–85.3
Cape Winelands	5	11.9	4–25.6	128	14.4	11.9–16.6	19	15.2	9.4–22.7
Eden	1	2.4	0.6–12.6	77	8.5	6.8–10.5	7	5.6	2.3–11.2
Overberg	0	-	-	31	3.4	2.3–4.8	0		
West Coast	0	-	-	18	2	1.2–3.1	1	0.8	0.2–4.4
Central Karoo	0	-	-	10	1.1	0.5–2	0		
**Rural**	6	14.3	5.4–28.5	264	29.2	26.2–32.3	27	21.6	14.7–29.8
Time on PI in years, median (IQR)	3.6	2.9–4.9	-	3.2	2.6–4.2	-	4	1.7–6.2	-
Total number of VLs whilst on a PI, median (IQR)	7	6–10	-	6	4–7	-	7	4–11	-
Total number of high VLs whilst on a PI, median (IQR)	6	5–7	-	5	3–6	-	6	2–9	-
Presence of 6-month dispensing gap	13	31	17.6–47.1	464	51.3	48–55	44	41.1	26.9–44.2
Presence of 12-month dispensing gap,	7	16.7	7–31.4	255	28.2	25.3–31.2	28	26.2	15.4–30.7
**Magnitude of third high VL**									
≥ 10 000 copies/mL	29	69	52.9–82.4	640	70.7	67.6–73.7	-	-	-
**Magnitude of last high VL**									
≥ 10 000 copies/mL	33	78.6	63.2–89.7	653	72.2	69.1–75.1	-	-	-
**Outcome of request for genotype test**									
Approved	37	88.1	74.4–96	-	-	-	92	73.6	65–81.1
Rejected	1	2.4	0.6–12.6	-	-	-	13	10.4	5.7–17.1
Provided	0	-	-	-	-	-	14	11.2	6.3–18.1
Not recorded	4	9.5	2.7–22.6	-	-	-	6	4.8	1.8–10.2

PHC, primary healthcare; IQR, interquartile range; VL, viral load; CI, confidence interval; PI, protease inhibitor; s.d., standard deviation.

### Step 3: Identification of predictors

In univariate analysis, several factors were associated with referral: receiving ART at a hospital rather than a primary healthcare (PHC) facility (odd ratios [OR] = 2.15, 95% CI 1.1–4.0), greater number of years on a PI (OR = 1.22, 95% CI 1.07–1.39), greater number of viral loads taken whilst on the PI (OR = 1.23, 95% CI 1.13–1.34) and greater number of high viral loads (i.e. ≥ 1000 copies/mL) whilst on the PI (OR = 1.37, 95% CI 1.18–1.59). Factors associated with non-referral were receiving ART care in a rural district (OR = 0.4, 95% CI 0.35–0.46) and the presence of a 6-month gap in dispensing (OR = 0.43, 95% CI 0.22–0.83).

In multivariate analysis, independent determinants of referral included receiving care at a hospital rather than a PHC facility (adjusted odd ratios [aOR] = 2.15, 95% CI 1.1–4.2), a higher number of VLs ≥ 1000 copies/mL whilst on a PI (aOR = 1.2, 95% CI 1.01–1.42) and a greater number of years on a PI (aOR = 1.25, 95% CI 1.07–1.46). Patients with a 6-month gap in dispensing were less likely to be referred (aOR = 0.37, 95% CI 0.17–0.81). The final model in the multivariate logistic regression analysis was highly statistically significant (*p* = 0.0000) (Table 3).

**TABLE 3:** Predictors of referral. Univariate and multivariate analysis.

Variable	Univariate analysis			Multivariate analysis (backwards stepwise, cut-off *p* > 0.05)		
	Odds ratio	95% CI	*p*-value	Adjusted odds ratio	95% CI	*p*-value
Age	0.99	0.97–1.03	0.870	-	-	-
Female sex	0.79	0.41–1.52	0.478	-	-	-
**Year of meeting referral criteria**						
2014	1	Reference	-	-	-	-
2015	2.11	0.63–7.08	0.226	-	-	-
2016	1.5	0.41–5.4	0.539	-	-	-
Facility type (hospital)	2.15	1.1–4.0	0.017*	2.15	1.1-4.2	0.025*
Rural	0.4	0.17–0.97	0.043*	-	-	-
Time on PI in years	1.22	1.07–1.39	0.002*	1.25	1.07–1.46	0.004*
Total number of VLs whilst on a PI	1.23	1.13–1.34	0.000*	-	-	-
Total number of high† VLs whilst on a PI	1.37	1.18–1.59	0.000*	1.2	1.01–1.42	0.040*
Presence of 6-month dispensing gap	0.43	0.22–0.83	0.012*	0.37	0.17–0.81	0.013*
Presence of 12-month dispensing gap	0.51	0.22–1.16	0.109	-	-	-
Third VL 1000–10 000 copies/mL	1.08	0.55–2.12	0.816	-	-	-
Last VL 1000–10 000 copies/mL	0.71	0.33–1.5	0.365	-	-	-

PI, protease inhibitor; VL, viral load; CI, confidence interval.

† , High VL defined as ≥ 1000 copies/mL.

* , *p* < 0.05.

### Did not meet criteria and referred

**FIGURE 4:**
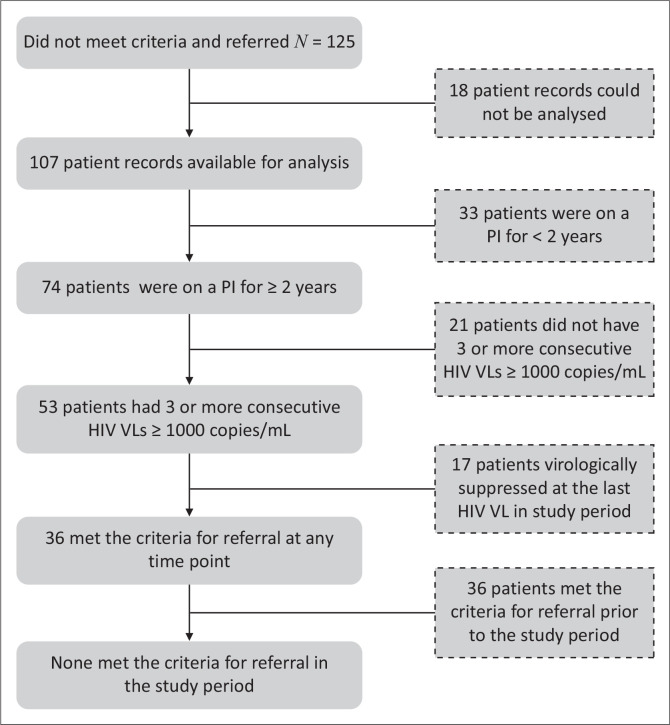
Further analysis: ‘Did not meet criteria and referred’ group.

Of the 125 patients who were referred despite not meeting the criteria for referral in the study period, 107 patient records were identified for further analysis using the folder number provided in the referral documentation. Where present, the folder number allowed for the identification of the PMI, which facilitated linkage with records in the study dataset. Figure 4 demonstrates the reasons why these patients were not included in the estimate of patients meeting the criteria for referral to the committee. An important finding is that 36 of the 125 patients in this group *did* actually meet the criteria for referral and that they were eventually excluded from the estimate because they had met the referral criteria prior to and not in the study period. The implication of this finding is explored further in the sensitivity analysis.

### Sensitivity analysis

A sensitivity analysis was undertaken because of two main concerns relating to the procedure used in Step 1 to derive an estimate of patients meeting the criteria for referral to the third-line ART committee in the study period. The first of these concerns related to the impact of patient adherence to therapy on the outcome, that is, referral. Adherence data were not captured by routine information systems and therefore could not be incorporated into the estimate. As clinical knowledge of adherence may impact on the referral decision, it is likely that the number of patients meeting criteria for referral was overestimated. In the sensitivity analysis, the presence of gaps between dispensing events was utilised as a proxy measure for non-adherence. Twelve and 6 month gaps in dispensing were considered.

The second concern pertains to the time period in which the criteria for referral were met. The study undertook to identify only those patients who had met the criteria in the period 01 October 2014 to 30 September 2016. However, prior to April 2013 no third-line ART referral system was available to patients failing second-line regimens. It is therefore plausible that patients who met the criteria for referral prior to the start of this period would be referred once the referral process and committee was established. This hypothesis is supported by the analysis of the ‘Did Not Meet Criteria and Referred’. For this reason, variation in the time period of meeting the criteria for referral was analysed. Patients meeting the referral criteria from 2012, 2013 and 2014 were considered.

In order to further the analysis, a combination of the two factors (i.e. adherence estimate and time period) was considered. For this analysis, we allowed for inclusion of patients meeting criteria for referral from 2012 until the end of the study period and excluded patients with a dispensing gap of 12 months. The resultant estimate was 1079 patients meeting the criteria for referral to the third-line ART committee.

Revision of the estimate improved the match of patients who met the criteria and were actually referred from 42 to 55; however, the number of patients meeting criteria and not referred was also substantially higher having increased from 947 to 1024 (data not shown). It is likely that the match may have been higher had actual referrals prior to 2013 been included.

In multivariate analysis, following a backward stepwise selection procedure with variables removed from the model at *p* > 0.05, only the number of VLs ≥ 1000 copies/mL whilst on a PI remained independently associated with the outcome, that is, referral. For each additional high VL, patients had 1.2 times the odds of being referred (95% CI 1.09–1.34, *p* = 0.000).

## Discussion

The study found that in 2 years, 947 patients met the criteria for referral to the Western Cape third-line ART committee – to be evaluated for genotype resistance testing as a precursor for consideration for third-line ART. This estimate, obtained from the analysis of routinely collected data, was far larger than the number of patients actually referred to the committee in the same period (*n* = 167). A comparison of the two groups identified only a small overlap in patients (*n* = 42) indicating both a large proportion of missed opportunities for referral and a considerable proportion of referrals that did not meet the referral criteria.

The study found that 6.9% (947 of 13 791) patients receiving second-line ART care for at least 2 years in the Western Cape should have been evaluated for third-line ART in a 2-year time period. As far as the author is aware, this is the first study to enumerate the need for third-line referral in this setting. This information is particularly useful for public health planning and may be considered a proxy for evaluation of second-line programmes.

The study found a poor match between individuals meeting the criteria for referral and those who were referred – indicating both a large number of missed opportunities and inappropriate application of the referral criteria. Missed opportunities for referral have considerable public health implications resulting in patients remaining on a failing regimen for a longer duration, accumulating further resistant mutations and potentially compromising future treatment options.^6^ In addition, delays in referral may facilitate transmission of resistant virus.

Comparative analysis identified that factors independently associated with referral included receiving care at a hospital rather than a PHC facility, receiving a PI for a greater number of years and having a higher number of viral load tests whilst on the PI. Patients who had at least a 6-month gap between dispensing records were less likely to be referred than those without.

In our study, for every additional year on a PI after the 2-year referral criterion, patients had 1.25 times the odds of being referred. One similar study in a South African cohort also identified that time on second-line therapy prior to the availability of third-line treatment was a strong predictor of switching to third-line therapy amongst patients with significant viraemia.^7^ Another study which looked at switching from first to second-line therapy found a decreased likelihood of switching in patients who started ART in a more recent calendar year.^8^ As clinicians are likely to first attempt adherence interventions prior to referral, this finding may reflect the referral of patients for whom adherence interventions were unsuccessful. We also found that the number of high viral loads whilst on the PI was independently associated with referral, indicating that even with the length of time receiving a PI held constant, patients who had more viral load tests done were more likely to be referred. Frequent viral load testing suggests clinician awareness of treatment failure and most likely reflects frequent review of adherence intervention efforts.

We also found that patients who were receiving care in a hospital were more than twice as likely to be referred than those who were receiving care at a PHC facility. This finding is not supported by a study analysing predictors of switching from first to second-line regimens, which did not find a significant difference in rates of switching between treatment providers, including providers in both hospital and PHC settings.^9^ However, variation in rates of treatment switch between clinics has been described previously.^10^ It is possible that the higher rate of referral from hospitals in our study reflects differences in the ART programme and patient profile in these settings. Patients in hospital-based ART centres may have more complicated treatment histories and may be more likely to be seen by doctors and therefore more likely to be referred. In addition, the higher proportion of referrals from hospitals may be masking prior up-referral from PHC to hospital-based ART centres because of treatment failure. Clinician awareness and compliance with the referral procedure has not been investigated; however, differences in clinician awareness and influence between these settings may also explain some of the findings.

Our study found that patient non-adherence (evaluated by the presence of a 6-month gap in dispensing as a proxy measure) resulted in patients being less likely to be referred. This finding is supported by others who have found that in the context of switching from first to second-line therapy, patients who missed visits^11^ and those who had no clinic contact for 4 months^12^ were less likely to switch from first to second-line ART.

Other studies looking at predictors of treatment switch have indicated magnitude of the viral load result as a significant factor, with values greater than log 4 more likely to result in switch.^11,12^ In our study, however, viral load magnitude at the third of the three high viral loads and at the last recorded viral load was not significantly associated with the referral outcome. Selection of these time points may explain why the study did not find an association between viral load magnitude and referral. It is possible that the findings may have differed if more sophisticated analysis were conducted by selecting the viral load result closest to the date of meeting referral criteria instead.

Parameters not evaluated in this study include CD4 count magnitude and rate of decline, which were found to be predictors of switching from a first to second-line regimen.^8,9,10,11,12^ In addition, the study did not investigate the impact of clinician knowledge of the referral criteria and process.

## Limitations

The estimate of patients meeting criteria for referral is subject to a number of limitations. Firstly, the estimate was derived from primary data sources, which may have been prone to data error. The TIER.net information system in particular is less robust than other sources of pharmaceutical dispensing data because of its reliance on manual data capture by information clerks. Unfortunately, TIER.net was a vital data source to the estimate enabling calculation of the duration of time an individual had been receiving a PI. Other sources of pharmaceutical dispensing data (JAC, CDU) have only been established in recent years and did not have wide facility and patient coverage. Secondly, there is potential for missed or incomplete linkage of records using the PMI.

Thirdly, whilst every attempt was made to model the estimate around the criteria for referral to the third-line ART committee, some parameters could not be established. For example, the referral criteria specified three consecutive high viral loads (≥ 1000 copies/mL) 8–12 weeks apart. The estimate was able to identify patients who had three consecutive high viral loads but did not determine the duration of time between each result. Furthermore, the three consecutive high viral load results may have occurred at any point in the patient’s history of receiving a PI, meaning that patients identified as meeting the criteria for referral may have achieved virologic re-suppression and therefore would not have been referred. In an attempt to mitigate this, the estimate excluded patients who achieved virologic suppression at the last recorded viral load in the study period.

The referral criteria also specified objective verification of adherence by pharmacy refill records and the completion of an adherence assessment. This could not be incorporated reliably into the estimate and was therefore excluded. This is likely to have resulted in an overestimate of patients meeting the criteria for referral. An attempt was made to address this limitation by undertaking sensitivity analyses, which looked at the impact of dispensing gaps (as a proxy for poor adherence) on the estimate. However, further analysis of patients who were actually referred revealed a high proportion of patients with dispensing gaps 12 months or longer. Based on the information at hand and because of the inability to validate the estimate it is unclear whether this finding relates to poor quality of the pharmaceutical dispensing data, which were utilised to determine gaps in dispensing.

Clinicians may have a better view of adherence than what can be gleaned from pharmaceutical records and may choose not to refer patients based on their assessment. In addition, whilst genotype resistance testing is not routinely available outside of this access programme, clinicians may have been able to access genotype testing via alternate means, for example, via research procedures – 8.4% of patients referred to the third-line committee did already have a genotype test at the time of referral (Table 1). It is possible that patients who might otherwise have been referred to the committee for genotype testing were not referred as a result.

An additional consideration (supported by both the sensitivity analyses and further analysis of the group of patients who were referred despite not meeting the criteria for referral) is that of a delay between meeting the criteria and actual referral. It is also important to acknowledge that referral of patients who did not meet the referral criteria may have been appropriate under certain special circumstances such as virological failure in pregnancy or virological failure because of incorrect PI dosing in the setting of concomitant tuberculosis.

The inability to validate the estimate is an important limitation of the study. No single data source could be used as a gold standard to verify the key clinical criteria utilised to derive the estimate. Folder review formed part of the initial study design for the purposes of validation and collection of additional data. However, following a pilot exercise, this aspect of the study was abandoned because of reasons of non-feasibility. This impacted both on the ability to verify the estimate and on the collection of data pertaining to explanatory variables.

Despite these limitations, this exercise demonstrated the use of routine data on a population level to identify patients requiring further intervention. In the future, widespread permeation of pharmaceutical information systems and improved data usage and quality may allow this exercise to prompt clinicians to refer patients appropriately. Similarly, this exercise may allow programme managers to evaluate the effectiveness of the second-line ART programme on provincial, district and sub-district levels.

## Conclusion

This study evaluated a third-line ART referral process in the Western Cape province of South Africa – the country with the largest ART programme in the world and the first public sector third-line access programme. This work adds to a limited body of knowledge pertaining to the need for third-line ART in South Africa, providing information that is valuable for public health planning and health programme evaluation.

The study identified missed opportunities for referral and inappropriate referral of patients to the third-line ART committee, which have significant public health implications.

The estimate of patients meeting the referral criteria was subject to a number of limitations and could not be reliably validated. However, with ongoing data usage, improved data quality and more sophisticated analysis, this method of identifying patients meeting the referral criteria could be used to prompt referral.

Future work should focus on refining the methods used to identify patients meeting the criteria for referral and determining the validity of the method. In addition, clinician awareness of the referral criteria and procedure should be evaluated to give a comprehensive view of the referral process and guide future intervention.
